# Hands Deserve Better: A Systematic Review on Surgical Glove Fit and Provider Performance

**DOI:** 10.3390/jcm14217695

**Published:** 2025-10-30

**Authors:** Abhishek Chatterjee, Deborah L. Spratt, Andreas Enz, Jessica Bah-Rösman, C. Tod Brindle

**Affiliations:** 1 Department of Surgery, Division of Plastic Surgery, Tufts Medical Center, Boston, MA 02111, USA; abhishek.chatterjee1@tuftsmedicine.org; 2 Independent Researcher, Hornell, NY 14843, USA; dspratt@rochester.rr.com; 3 Department of Orthopedics, Helios Kliniken Schwerin, Medical School Hamburg, 19055 Schwerin, Germany; 4 Department of Orthopedics, University Medicine Rostock, 18057 Rostock, Germany; 5 Global Medical Affairs, Mölnlycke Health Care, 43121 Mölndal, Sweden; jessica.bahrosman@molnlycke.com; 6 Independent Researcher, Norcross, GA 30092, USA; c.todbrindle@gmail.com

**Keywords:** glove fit, surgical site infection, gloving practice, surgical gloves, glove breach

## Abstract

**Background:** The maintenance of an aseptic barrier between the surgical team and patient aids in the prevention of exposure of the patient to pathogens. Variations in gloving practice may have safety implications due to glove failure. An important relationship exists between optimum glove fit and manual dexterity, tactile sensitivity, and fatigue. The aim of this systematic review was to assess the presence and quality of the available literature that investigates the critical association between glove fit and provider performance in the operating theatre and to ascertain whether there is an established standard to determine appropriate glove size. **Methods**: A systematic review of the literature was undertaken in accordance with the PRISMA statement using one distinct research question regarding glove fit (INPLASY2025100008). Searches on PubMed, Embase, Cochrane Collaboration of Systematic Reviews and Metanalyses and Google Scholar were performed between 1 May 2022 and 24 January 2023. Studies were assessed for eligibility against pre-defined inclusion and exclusion criteria. Risk of bias was determined using multiple assessment tools. **Results**: This systematic review included 18 studies, nine of which were high-quality Level I or II trials, and multiple observational analyses. Poor glove fit was consistently associated with reduced manual dexterity, impaired tactile sensitivity, and decreased comfort, while oversized or undersized gloves increased the risk of glove perforation and fatigue. These findings underscore the clinical importance of appropriate glove sizing to optimize surgical performance and safety. **Conclusions**: There is a scarcity of high-quality studies investigating the relationship of glove fit and performance. Furthermore, there does not appear to be a standard method to determine the optimal glove fit for members of the surgical team, nor are there practical examples of how glove size is determined. Further research in this area is required.

## 1. Introduction

Surgical gloves were introduced in the late 19th century to protect both patients and providers, and their use rapidly evolved with improvements in material technology. Modern glove innovations, such as latex-free and polyisoprene products, have focused on balancing tactile sensitivity with safety and comfort. Despite this evolution, the importance of proper glove fit remains underexplored, even though ergonomic discomfort and fatigue are well-documented contributors to occupational injury among surgeons.

In a nationwide survey of US surgeons (*n* = 2295) conducted in 2019, musculoskeletal disorders of the back, hands, lower extremities, and neck were the second most reported reason for retirement after advancing age [1]. A quantitative cross-specialty survey of UK surgeons assessed the prevalence of musculoskeletal disorders over all anatomical zones. 86.5% of respondents had reported some musculoskeletal discomfort in the previous 12 months, with 92% reporting an issue over the previous five years. 86% of respondents felt that their symptoms were related to posture at work, with 63% stating an impact on their home life and 37.5% stating that it altered or stopped their work [2]. Further, a study of orthopedic surgeons in Singapore observed that there was a high prevalence of musculoskeletal symptoms, particularly in the neck (66.1%), the hands and wrists (42.9%), and the shoulder (39.3%) [3].

**Figure 1:**
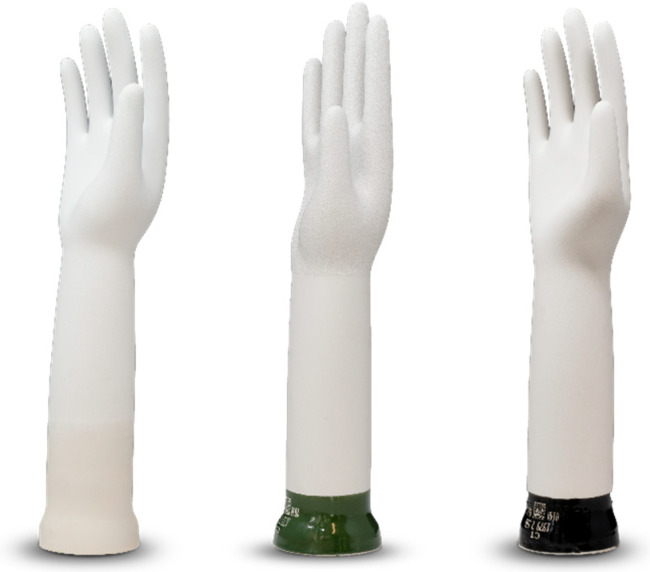
Glove Formers. Glove formers from a single manufacturer. Note the anatomical differences in size, width and shape of different formers: left–curved; middle–straight; right–custom proprietary (Image used with permission from Mölnlycke Health Care AB. © 2025 Mölnlycke Health Care AB. All rights reserved).

Interestingly, the average healthcare provider is often unaware of the unique differences in the construction of surgical gloves and, more importantly, how this may impact potential performance. The idea that a “glove is a glove” inaccurately leads the provider to a glove selection that may not be optimized to their practice, surgical specialty, or unique anatomical and gender-specific needs. Procurers making product decisions based on the idea that “all gloves are created equal” may impact providers due to significant differences between manufacturers and their respective manufacturing processes, raw materials, and formers. For example, formers used to shape gloves during the dipping process may be classified as “straight”, “curved”, or a specialized, proprietary former (Figure 1). The impact of the baseline anatomical position of the fingers and thumb, as well as the variation between the specific dimensions of palm, finger, and wrist sizes between the former and the actual user, necessitate that healthcare personnel have a reliable method to ensure appropriate fit during a procedure. Factors such as glove resistance, constriction, and fit during repetitive motions or surgical tasks may lead to challenges in ergonomics, fatigue, and quality of life. Therefore, providers should consider how they will determine appropriate glove style, type, and sizing based on the variety of available manufacturers.

The complex anatomical structure of the hand allows for a variety of possible functions with highly detailed capabilities. Hand performance has been described as the ability to perform a variety of skills including grasp, muscle strength, movements, tactile sensitivity, and motor coordination [4]. Dianat and colleagues explored the impact of protective gloves on these movements in a systematic review of methodologies to test glove impact on manual dexterity, handgrip strength, tactile sensitivity, range of finger and wrist movements, muscle activity and fatigue. The review identified that gloves may reduce manual dexterity, tactile sensitivity, and handgrip strength while increasing the potential for fatigue. However, these results were impacted by the different types of gloves tested in these studies. Glove research has previously identified that glove type directly impairs manual performance in industrial and military personnel [5]. Chang and Shih demonstrated the relationship between hand dimension changes after glove application on the muscle-tension relationship. Their work studied ten female volunteers with four different cloth glove variations and showed that as the number of gloves increased, the grip force decreased, yet fatigue was not significantly different between groups [6]. In dental practice, a prospective observational study comparing non-sterile medical gloves of vinyl versus latex composition indicated that both the material and the degree of appropriate fit impacted the perceived tactile discrimination of the participants [7]. Interestingly, while a study of volunteer health care workers demonstrated via peg-board test that surgical gloves that were too small or too large altered performance compared to “right-size” gloves, the selection and sizing of what was considered right-sized was based on the volunteer’s existing experience and not a formal assessment [8]. With the variability in manufacturer glove sizes, shapes, and materials, understanding how clinician performance is impacted by glove selection and the relative method by which the gloves were sized to the individual remain unknown.

Recent studies have also shown that surgical setting and procedure type can influence glove integrity and performance. For example, a systematic review comparing unnoticed glove perforations in minimally invasive versus open surgeries demonstrated that the surgical environment significantly affects glove durability and safety for both providers and patients [9]. In order to examine the relationship between glove fit and provider performance, a systematic review of the literature was performed.

The objective of this project was to determine the best available evidence describing four key fundamental principles of surgical gloving practice: glove fit, double gloving, puncture indication, and glove change frequency. The purpose of the research and consensus process is to inform existing and future operating room staff on the importance of appropriate gloving practice to optimize healthcare provider (HCP) performance and ensure provider and patient safety.

This article specifically reports on the glove fit component of this comprehensive systematic review of the literature with the following research question: For healthcare providers scrubbed into surgical procedures, what is the association between poor glove fit and provider performance in the OR? A secondary aim of this research asks, is there an established standard to determine appropriate glove size?

This systematic review was one arm of a 4-arm, parallel, systematic review of the literature and Delphi-consensus recommendation project on surgical gloving practice [10].

## 2. Materials and Methods

The study was conducted in accordance with preferred reporting items for systematic reviews and meta-analysis protocols (PRISMA) statement and follows the published checklist for reporting (Table S1) [11]. The protocol for this study was registered on the International Platform of Registered Systematic Review and Meta-Analysis Protocols (INPLASY; registration number INPLASY2025100008).

### 2.1. Eligibility Criteria

This systematic review was performed between 1 May 2022 and 24 January 2023. The objective of this study was to determine the best available evidence describing the association between surgical glove fit and health care provider performance during surgical cases in the operating theatre. An ethnographic research study of healthcare providers indicates that surgical gloves are perceived as a commodity across a variety of manufacturers and product types [12], and therefore individual surgical glove characteristics such as former type used, size, raw material, resting position, resistance against movement and durability are often not considered relative to HCP experience during surgery. The purpose of the study was to help inform current surgical praxis and future innovation to deliver optimal hand performance.

Therefore, the research question for this systematic review was: What is the association between poor glove fit and provider performance in the OR? A secondary aim was to ascertain whether there is an established standard method to determine appropriate surgical glove size for health care providers.

To meet the objectives and research question, a pluralistic approach was used to identify all available evidence and determine the highest-quality evidence. 1980 was used as the cut-off point for including historical studies, as latex surgical gloves were first introduced to the market in 1964, with significant advances in present day raw materials introduced in the late 1990s (polyisoprene) and into the 2000s (polychloroprene). However, 1980 was used to avoid historical bias associated with the HIV/AIDS epidemic, which brought considerable attention to personal protective equipment and the performance of surgical gloves as an essential barrier in the operating room. While advances in the field are ongoing for manufacturers of surgical gloves, articles were considered if they were published between 1 January 1980 through to 1 January 2023. Study inclusion hierarchy was purposefully broad to capture bench testing, healthy volunteer and clinical studies given the nature of the evidence gap and potential for subjectivity in provider experience. Therefore, the hierarchy was inclusive of:

- Systematic reviews & meta-analysis
- Randomized Controlled Clinical Studies
- Quasi-experimental studies
- Cohort with control designs
- Case controlled designs
- Observational studies
- Quality improvement projects/gray literature when demographics, methods and outcomes are clearly reported
- Mixed methods approaches
- Qualitative surveys
- Healthy volunteer studies
- In vitro studies

Studies were allowed to report on the association of glove fit and provider performance in laboratory-simulated surgical settings, or surgical procedures within adult and pediatric populations, across any type of surgery, and in acute-care hospital operating rooms. Studies were included if they had English abstracts; however, there were no language exclusions for full-text review. All articles were translated to English for use by the reviewers with native language capability or using third-party translation software.

Studies were excluded if they reported testing of non-sterile medical exam gloves, or if they involved dental or orthodontic procedures performed outside of an operating theatre setting. These were excluded because their procedural environments, glove use patterns, and ergonomic demands differ substantially from those of sterile surgical practice, and their inclusion could have introduced heterogeneity unrelated to intraoperative performance. Similarly, ‘antimicrobial glove’ studies were excluded as they primarily assess microbiological efficacy or barrier integrity of coated materials rather than the impact of glove fit on provider dexterity or comfort.

### 2.2. Information Sources

Four databases were used to identify prospective articles, including PubMed, Embase, Cochrane Collaboration of Systematic Reviews and Metanalyses, and Google Scholar. The primary search was conducted in November 2022, independently by two reviewers (AC and DS), with monthly verification by a third reviewer (AE). Secondary manual searches of reference lists were also performed to ensure completeness. All non-open access articles were purchased through RightFind Articles.

### 2.3. Search Strategy

**Figure 2:**
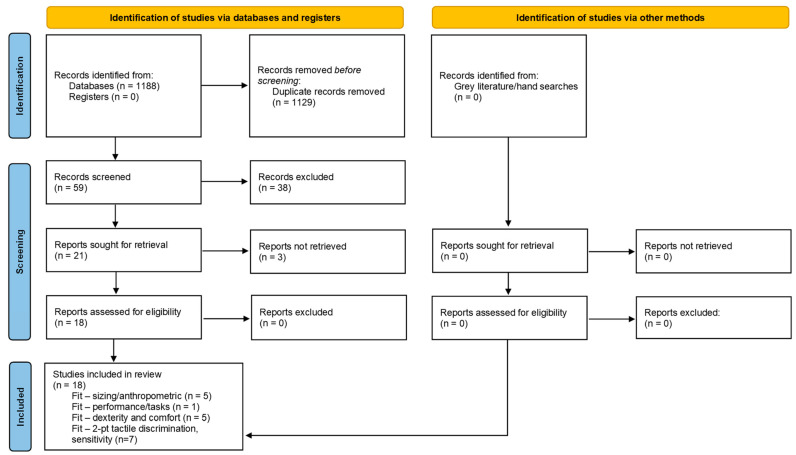
PRISMA Flow Chart.

The search strategy was defined through combination of four domains and initial key terms, with a second selection of terms added following initial search results (Table 1). Domain one defined terms for the population and setting, domain two identified terms associated with the device (glove), domain three explored relevant terms the concept of glove fit and domain four covered provider performance. Four weeks of initial keyword searches were performed to help refine the final study keywords to improve precision of abstract identification. The primary search limits for the databases included a custom date range of 1980–2023. Search limitations including Boolean phrase NOT terms were selected based on initial keyword search findings. Results of the search strategy will be reported using the PRISMA study flow chart (Figure 2).

### 2.4. Data Management and Selection Process

Two reviewers (AC & DS) independently provided screening of all identified abstracts for eligibility. Critical appraisal was carried out by monthly meetings to verbally discuss inclusion versus exclusion designation. When there were disagreements with any aspect of the process regarding identified studies, the reviewers presented their rationale for selection and determined if an agreement could be made on inclusion. If agreements could not be made, a third researcher (AE) would be called in to render a final decision. The reviewers used an encrypted sharing platform via Microsoft Teams which was only accessible by invitation from the reviewers. Following agreement on which abstracts to send for full review, the reviewers independently read all full-text manuscripts and subsequently met to determine final studies for analysis. Meta-analysis was not a goal of this systematic review due to expected methodological heterogeneity among the studies. Given the broad inclusion criteria and variation in study scope, data acquisition, assessment and reporting, meta-synthesis of study outcomes was utilized. The data collection process for extraction of each study was performed by the researchers in tandem over multiple virtual and in-person meetings where data reporting was categorized by outcome domains and transferred along with study demographics into evidence tables.

### 2.5. Risk of Bias

Risk of bias was assessed using validated tools appropriate to study design: the Newcastle–Ottawa Scale (NOS) for non-randomized studies [13]; the Cochrane Risk of Bias 2.0 (ROB 2.0) for randomized-controlled trials [14]; and the ROBIS 1.2 tool for systematic reviews [15]. Each study was independently assessed by two reviewers (AC, DS). Discrepancies in scoring were discussed and resolved by consensus, with arbitration by a third reviewer (AE) when required. Quality categories (‘high’, ‘low’, ‘some concerns’) were assigned according to established thresholds of each tool.

### 2.6. Data Synthesis

Because of the heterogeneity in outcome definitions across studies—ranging from manual dexterity, tactile sensitivity, and knot-tying ability to comfort, fatigue, and glove integrity—a formal meta-analysis was not feasible. Therefore, outcomes were systematically categorized into four domains for structured narrative synthesis: (1) manual dexterity and psychomotor performance, (2) tactile sensitivity and discrimination, (3) comfort and fatigue, and (4) glove integrity and perforation risk. Findings within each domain were summarized descriptively according to study design, level of evidence, and direction of effect.

### 2.7. Meta-Bias

Given the variation of possible study designs and outcomes, traditional use of critical appraisal tools was replaced with monthly discussions by the reviewers to examine inherent bias based on included articles and their respective designs, whether they were systematic reviews or otherwise.

### 2.8. Confidence in Cumulative Evidence

Overall confidence in the quality of study evidence and recommendations for clinical practice will be evaluated using the Johns Hopkins Nursing Evidence-Based Practice Appendix C: Evidence level and quality guide [16].

## 3. Results

Figure 2 provides the PRISMA 2020 flow diagram illustrating study selection. A total of 1188 records were identified through database searching, and no additional records were identified through manual searches. After removal of duplicates (*n* = 1129), 59 unique titles and abstracts were screened according to the inclusion/exclusion criteria. A total of 38 studies were excluded from further analysis and 3 were unable to be retrieved, leaving 18 records assessed for eligibility for full text review. All 18 studies were determined to be eligible for inclusion in the review (Table 2 and Table 3). Level of evidence, quality assessments and risk of bias scores are detailed in Table 4.

### 3.1. Summary of Evidence Prior to Current Review

No prior Cochrane review or systematic reviews specifically addressing glove fit were identified during the literature search. Comprehensive Cochrane reviews on gloving have addressed the issue of glove perforation regarding double gloving and glove thickness, concluding that double gloving reduces the risk of exposure to the surgical team, without significantly compromising on tactile sensitivity [32,33].

### 3.2. The Effect of Glove Fit on Surgical Manual Dexterity, Tactile Sensitivity and Performance

Three level IA studies and one IIB study demonstrate differences in tactile sensitivity, two-point discrimination (2PD) and dexterity or task performance between manufacturers’ gloves and within-manufacturer glove styles (Table 2) [8,21,22,23].

Man and colleagues (2022) conducted a level IA cross-sectional, single-center pilot study, where surgical team members were tested for manual dexterity using the 2PD and Semmes–Weinstein mono-filament testing (SWMT) of both index fingers. In surgeons, the undersized gloves resulted in significantly better 2PD results than the use of well-fitted gloves. The SWMT assessment also resulted in improved scores for undersized gloves compared to well-fitted gloves, although this was not statistically significant. The authors propose that smaller gloves result in a thinner material covering as it is stretched over the fingertips. However, surgical team members reported higher discomfort with the use of undersized gloves. Other surgical team members did not show any change in 2PD with either over or undersized gloves. The use of oversized gloves resulted in a deterioration of dexterity as measured by the SWMT test in all team members assessed [23]. The second IA study, a single-blind RCT crossover study by Hardison et al. (2017) [21], investigated the ability of 41 medical and dental students to perform simulated microsurgical tasks whilst wearing double gloves. A statistically significant increase in the time taken to perform the microsurgical task was observed as the thickness of the gloves increased [21]. In a level IA crossover randomized trial, Johnson and colleagues (2013) used psychomotor aptitude tests (SWMT, Crawford Small Parts Dexterity Test (CSPDT), and PPT) to determine whether glove use impacts tactile and psychomotor performance compared to bare hands. The authors reported that sensation was reduced overall by the wearing of gloves. Statistical differences were observed for glove comfort ratings across all the types of gloves tested, but these did not correlate with performance ratings. Use of the thickest glove assessed in this study resulted in the slowest performance times [22].

In a level IIB observational study, Drabek and colleagues evaluated the impact of preferred-sized gloves while performing a peg board insertion test with gloves that were one full size smaller and larger. The level II study indicated that wearing gloves too small or too large increased the time to complete the tests by 7–10%, respectively (*p* < 0.05; *p* < 0.001). The smaller gloves limited hand motion and caused discomfort, while larger gloves were reported as being clumsy but comfortable [8].

Three level III observational studies support the importance of glove selection on surgical performance, with two studies showing that glove thickness has an impact on touch sensitivity and manual dexterity [5,26] and one demonstrating that 2PD measurements improve over time as surgeons adapt to double gloving/glove fit [27].

A level III study with good quality and high risk of bias compared the manual dexterity and performance of 12 men utilizing three different thickness levels of gloves (0.18 mm, 0.36 mm, and 0.64 mm). The tests were performed over 14 sessions on consecutive days. Initial results demonstrated a decreasing performance speed compared to bare hands and a worsening of this observation with increased thickness of gloves. Linear regression showed that performance times had a linear increase to time of completion based on glove thickness. However, performance with the gloves improved and, in some cases, surpassed times of the earlier test, indicating adaptation of performance to glove thickness over time. Additionally, the rate of glove damage was proportional to glove thickness over the course of the study, with thicker gloves demonstrating less glove damage [5].

### 3.3. Glove Fit and Comfort

Two level I and two level II observational studies report evidence that glove fit impacts surgical team members’ comfort (Table 2). A level I randomized study (low quality, some bias) by Wilson et al. (1996) [24] investigated the selection of gloves when double gloving and determined that the use of a larger pair of gloves inside a usual sized outer glove was the combination preferred by most surgeons when two pairs of gloves are worn. This combination led to improved comfort, sensitivity, and dexterity, although this was not statistically significant [24]. A further level I study by Man et al. (2022) reported that the surgical team experienced discomfort and pain when using undersized gloves and that oversized gloves were the most comfortable [23].

In the first of the level II studies, Drabek et al. (2010) [8] observed that subjects reported that undersized gloves constrained movement and were painful. In contrast, gloves that were too large resulted in extra material in the fingertips and a loss of tactile sensitivity [8]. Bucknor and colleagues (2011) [18] reported in a level II observational study that there was no difference in tactile performance, as measured with the 2PD test, between a gloved hand and bare skin. The authors concluded that there are high levels of tactile feedback designed in modern glove technology and that surgeons may prefer one type of glove over another based on ‘feel’ [18].

Multiple level V studies further report on the importance of glove fit and comfort with evidence that poorly fitting gloves impact repetitive strain injury due to vascular obstruction [28] and highlight the importance of glove selection for fit and comfort when the surgical practice of double gloving is used [27]. Studies suggest the need for the manufacture of gloves with variable finger lengths for optimum fit and that although hand circumference is correlated with preferred glove size, hand anthropometry should be used in the future design of gloves for optimal fit [29,31].

### 3.4. Associations of Glove Fit with Glove Integrity

One level IIB study demonstrates the impact of glove fit on glove perforations, while one IIC study discusses the impact of glove hydration on glove fit (Table 2) [17,20].

The level IIA study by Gunasekera et al. (1997) [20] assessed the rate of perforation in gloves used in elective gynaecological surgeries during a five-month period. The procedures lasted more than 30 min and blood loss of more than 100 mL was observed. Perforations were detected by filling the glove with water and tying the cuff before squeezing the glove to observe water loss. 654 gloves were studied in total, and during the study, members of the surgical team had to use size 7 ½ gloves instead of size 6 ½ to 7, due to supply issues. When the correct size gloves were not available, the perforation rate of the gloves increased significantly for all team members. The overall observed rate of perforations rose from 81.8% to 100%. When well-fitting gloves were used, the perforation rate decreased to 71% [20].

Aggarwal et al. (1994) [17] conducted a level IIC study that examined the impact of glove hydration that occurs over the course of a surgical procedure, and is termed ‘glove growth’, on the fit of surgical gloves. Gloves were selected that fit subjects well at the beginning of the study and the fit was re-examined after submersion of the hands in water for 60 mins. Fit was assessed by the detection of creases or wrinkles. ‘Glove growth’ due to glove hydration compromised fit and induced increased creases and wrinkles [17].

## 4. Discussion

This systematic review identified a relative lack of studies with sufficient quality to render conclusions on glove fit and performance. The lack of sufficient quality ranges from the majority of studies having a low level of evidence (Level V case reports/series) or study designs that did not address standardized scientifically methodology on determining fit and how that effected performance. Performance also had a varied definition depending on the specialty being studied, understanding that laparoscopic performance can be different to how orthopedic surgeons define performance. The wide range of study designs and associated bias highlights the need for more research in this area. The review identified a randomized controlled trial and observational data that demonstrate the diversity of gloves available to the surgical team and the differences that are observed in the areas of tactile sensitivity, manual dexterity, comfort, and perforation rate. Future studies need to be speciality-specific, define how they address “good versus poor” fit and come up with meaningful performance outcome measures that may include measures based on successful task outcomes in addition to comfort and fatigue.

Manufacturers utilize different sized formers (Figure 1) (a hand-shaped cast to dip into glove components such as latex) in a variety of anatomical positions, which may impact individual glove fit. For example, straight versus curved formers may impact the natural resting position of the hand and provide varying levels of resistance to hand mobility. The impact of these differences may be subtle yet compounded over hours of surgery and repetitive movements, such as the tying of knots during suturing.

Gloves that are undersized tend to be painful to the wearer and are likely to increase fatigue [4,23]. Perhaps surprisingly, some studies have shown that undersize gloves may improve the tactile sensitivity of the wearer, likely due to a thinning of the material at the fingertip [23]. However, if undersized gloves are more likely to cause pain/fatigue, this slight improvement in tactile sensitivity should be weighed against the disadvantages of use. Extra-thin gloves are available from manufacturers and may benefit specific surgical specialties where a high level of tactile sensitivity and manual dexterity is required, while thicker gloves may be appropriate in surgeries where a higher level of surgical protection is of concern [5]. A systematic review by Enz and colleagues (2024) investigating the rate of glove damage during surgery and its relationship to glove change frequency demonstrates a high rate of unidentified perforation across multiple surgical specialties, highlighting this issue [10].

Oversized gloves, while in some cases more comfortable for the surgical team members to wear, may result in an increase in observed glove perforation rate and a decrease in manual dexterity [8,20,23].

The current findings have practical relevance for surgical practice and hospital procurement. In everyday clinical settings, they support the implementation of routine glove fitting assessments and highlight the importance of offering a wide range of sizes and fits to accommodate individual surgeon needs. Furthermore, procurement policies should prioritize evidence-based glove performance characteristics rather than cost alone, as appropriate glove selection can reduce fatigue, improve dexterity, and lower the risk of unnoticed glove perforations—ultimately enhancing intraoperative safety for both the surgical team and the patient.

Therefore, glove fit is an important factor to consider for optimal surgical performance in the operating room as poorly fitting gloves can be detrimental to both safety and performance measures. 

### 4.1. Opportunities for Future Research

At the time of writing, there does not appear to be a standard method to determine the optimal glove fit for surgical team members, nor are there practical examples of how glove size is determined. There is a need for glove manufacturers to design gloves with variable finger lengths, perhaps designed using anthropomorphic measurements, and for surgical team members to undergo specific glove fitting sessions prior to surgery to ensure optimal glove fit.

Interestingly, female surgeons suffer from a high rate of forearm, hand, and wrist pain across various specialties, with 68.3% of female arthroplasty surgeons reporting occupational injuries in these areas [34]. A study of 329 Australian surgeons indicated that female sex was a significant predictor of pain (*p* < 0.001), with 76% of female surgeons reporting neck, shoulder, and upper back pain in a 12-month period [35]. One hypothesis from authors both in orthopedic and laparoscopic surgery is that the instruments themselves may be designed for larger hand sizes, putting surgeons (and possibly female surgeons) at greater risk of musculoskeletal disorders due to the need for more grip strength and manipulation of instruments not designed to their specifications [36,37].

Research is needed to determine gender-related differences in palm width, finger length, wrist circumference, and associated measurements that influence glove fit. As most gloves are made from a standard former/cast, gender-specific measurements are required to inform manufacturers in the production of gloves with appropriate dimensions for fit and performance across the surgical team.

### 4.2. Limitations

Limitations of this review include the observational, single-center nature of many of the included studies, resulting in generalized findings. There is a need for future research on the issues surrounding optimal glove fit.

## 5. Conclusions

This systematic review highlights the lack of high-quality evidence on the relationship between surgical glove fit and performance. Existing studies are often limited by low levels of evidence, inconsistent definitions of performance, and non-standardized methods for assessing fit. Findings suggest that poorly fitting gloves—whether undersized or oversized—can negatively affect manual dexterity, tactile sensitivity, comfort, and safety. Given the potential impact of glove design, material thickness, and hand anatomy on surgical performance and occupational health, there is a clear need for standardized, specialty-specific research and manufacturer collaboration to develop gloves that optimize both fit and function for diverse surgical teams.

## Figures and Tables

**Table 1:** Domains and keywords.

Domain	Stage 1 Terms	Stage 2: Expanded Terms Based on Initial Search
Domain 1: Population/setting	Operating theatre Operating room Operation Surgery Surgical Surgeon Hospital Acute care	Procedure Perioperative Scrub tech 1st Assist Scrub Nurse Nurse
Domain 2: Glove	Surgical glove Sterile glove Procedural glove Attire	Latex Polyisoprene Polychloroprene
Domain 3: Fit	Fitting Right Suitable Adequate Proper Size Sizing	Hand-size Magnitude Dimension Range Scope Palm size
Domain 4: Performance	Indicator-system Identifier Awareness Detection Visual Exposure Recogni * Reveal See	Ergonomics Comfort design Functional design Human factors
Identified exclusion topics	Exam glove Clean glove Vinyl Nitrile	Condom

* indicates search screen wildcard to search for alternate spellings such as recognize and recognise.

**Table 2:** Level I/II studies.

Author, Year	Title	Aim	Key Findings
Aggarwal, M. et al., 1994 [17]	Biomechanics of surgical glove expansion	Determine if glove hydration alters biomedical performance of latex gloves Assess “glove growth” (swelling of gloves over time)	2.5 cm increase in length of dorsal surface of latex glove after flexion of index finger Significantly irreversible elongation and creep strain in one glove tested with evidence of ‘glove growth’, development of wrinkles and irreversible damage
Bucknor, A. et al., 2011 [18]	A comparison of the effect of different surgical gloves on objective measurement of fingertip cutaneous sensibility	Compare 2 different manufacturers on 2PD 52 (surgeon/RN/MD/Med student)	Bare skin was significantly more sensitive than wearing gloves; no differences observed in sensitivity between gloves. No statistically significant differences in 2PD were observed between gloves and bare hands.
Carter, S. et al., 1996 [19]	Can surgical gloves be made thinner without increasing their liability to puncture?	Assessment of glove durability comparing an existing product with a newer product 21 surgeons; 2PD; knot-tying; comfort; surgery: 280 cases	Thinner gloves scored statistically better in 2PD No difference was observed in knot tying Thinner gloves were more comfortable No differences in perforations
Drabek, T. et al., 2010 [8]	Wearing the wrong size latex surgical gloves impairs manual dexterity	Assessment of poorly fitting gloves on the impairment of manual dexterity. Grooved pegboard test to 20 healthy volunteer HCPs 3 runs each: bare hands, preferred size, full size smaller; full size larger	No difference between bare hands and preferred size Significant increases in peg insertion times with incorrect sizes size smaller = painful and limited hand motion; size larger = increased clumsiness but comfortable.
Gunasekera, P.C. et al., 1997 [20]	Glove failure: an occupational hazard of surgeons in a developing country	Determine the incidence of glove tear or puncture after surgery Ascertain what percentage of ‘unused’ gloves (i.e., previously used and autoclaved) remaining at the end of an operating theatre session were punctured	The perforation rate was 194/654 (29.6%). Coincidentally, well-fitting gloves were unavailable during part-1 of the study. The puncture rate was significantly higher (*p* < 0.01) during this period. Defects were found in 12.75% of gloves prepared for reuse. The rate of perforations was 72/88 (81.8%). This increased to 32/32 (100%) when ill -fitting gloves were used, compared with 40/56 (71%) when the proper sizes were available.
Hardison, S. A. et al., 2017 [21]	The effects of double gloving on microsurgical skills	Does DG negatively impact ability to perform simulated microsurgical task? Single blind RCT crossover 41 medical and dental students Simulated insertion of stapes prosthesis into ossicular model under microscopy	Outcome of interest: time to complete task during fine motor skills. No gloves were better than both experimental groups No difference between gloving groups statistically significant increase in the time taken to perform microsurgical task as thickness of gloves increased
Johnson, R. L. et al., 2013 [22]	Factors that influence the selection of sterile glove brand: a randomized controlled trial evaluating the performance and cost of gloves	RCT evaluating whether glove use impacts tactile & psychomotor performance compared to no glove (SWMT, CSPDT and PPT) Evaluate factors influencing selection of sterile glove brand 42 participants (based on sample size calc) (Anaesthesiologists, CRNAs, Residents, sCRNAs).	Tactile sensation reduced by wearing gloves compared to bare hands Statistically significant differences were observed for glove comfort across range tested, but this did not correlate with performance ratings. The thickest glove resulted in the slowest performance times.
Man, T. et al., 2022 [23]	Surgical experience and different glove wearing conditions affect tactile sensibility	Determine whether there is a difference in tactile sensitivity when comparing experienced surgeons with non-surgical staff N-54; 27 surgeons/27 medical students and nurses Analysed 6 glove brands with: SG, DG, regular size vs. oversized and undersized gloves Tests: SWMT; 2PD	Improved tactile performance of surgeons and non-surgeons wearing undersize gloves, but discomfort noted Oversize gloves decreased SWMT and 2PD when used by surgeons Surgical experience improves tactile sensitivity. Surgeons 2-PD testing results superior to non-surgeons.
Wilson, S. J. et al., 1996 [24]	Subjective effects of double gloves on surgical performance	Compare comfort, sensitivity and dexterity of SG and DG Assess different combinations of DG: A = SG; B = inner-normal, outer-0.5 size larger; C = inner-larger, outer-normal and D = DG normal size	32 surgeons, 384 cases or 96 sets (of 4 glove sets) Only Group A vs. B, C, D were significant. No difference b/w group B vs. C vs. D Between DG groups, C was superior to group B and D

2PD = two-point discrimination; CRNA = Certified Registered Nurse Anaesthetist; CSPDT = Crawford Small Parts Dexterity Test; DG = double gloving; HCP = HealthCare Professional; MD = Doctor of Medicine; PPT = Purdue Pegboard Test; RCT = Randomized Controlled Trial; RN = Registered Nurse; SG = single gloving; SWMT = Semmes–Weinstein Monofilament Test.

**Table 3:** Level III/V studies.

Author, Year	Title	Key Findings
Basak, T. et al., 2022 [25]	Comparison of surgical gloves: perforation, satisfaction and manual dexterity	Scrub nurses found powder- and latex-free gloves more comfortable to wear than latex and powdered gloves.
Bensel, C. K. 1993 [5]	The effects of various thicknesses of chemical protective gloves on manual dexterity	Performance in manual dexterity tests is best with bare hands and decreases with increasing thickness of glove.
Brunick A.L. et al., 1990 [7]	Comparative study: the effects of latex and vinyl gloves on the tactile discrimination of first year dental hygiene students.	No difference in tactile discrimination between vinyl and latex gloves. Subjects’ perception that latex gloves improve tactile sensitivity but may have been due to feeling of improved fit compared to vinyl gloves.
Kopka, A. et al., 2005 [26]	Anaesthetists should wear gloves--touch sensitivity is improved with a new type of thin glove	Extra-thin latex gloves showed increased touch sensitivity, as measured using a modified von Frey hair test, compared to standard latex gloves.
Novak, C. B. et al., 1999 [27]	Evaluation of hand sensibility with single and double latex gloves	Statistically significant difference between no gloves vs. gloves and single vs. double glove using SWMT and moving 2-PD. Suggest when double gloving that different combinations of fit are tested by surgeon for optimum dexterity.
Powell, B. J. et al., 1994 [28]	Evaluating the fit of ambidextrous and fitted gloves: implications for hand discomfort	Ambidextrous gloves exert a statistically significant larger force than fitted gloves. Increased pressure over time could contribute to muscle fatigue.
Rudolph, R. et al., 2022 [29]	An Unmet Need - Surgical Gloves with Variable Finger Lengths	Variable finger length observed in size 7.5 gloves from different manufacturers. A high percentage of respondents want gloves with better finger fit. Manufacturers should reassess surgical glove design.
Stellon, M.A. et al., 2016 [30]	The effect of surgeon hand anthropometry on surgical glove sizing and implications (Master’s thesis)	Anthropomorphic hand measurements were taken and correlated to surgeons preferred glove size. Showed statistically significant difference between surgeon hand size and general population. Showed hand circumference drove size choice.
Stellon, M. et al., 2017 [31]	Assessing the Importance of Surgeon Hand Anthropometry on the Design of Medical Devices	Anthropomorphic hand measurements were taken and correlated to surgeons preferred glove size. Showed statistically significant difference between surgeon hand size and general population. Showed hand circumference drove size choice.

2PD = two-point discrimination; SWMT = Semmes–Weinstein Monofilament Test.

**Table 4:** Level of evidence, quality and risk of bias assessments.

Author, Year	Level of Evidence	Quality	Risk of Bias
Aggarwal, M. et al., 1994 [17]	Level II	Low	High
Basak, T. et al., 2022 [25]	Level III	Low	High
Bensel, C. K. 1993 [5]	Level III	Good	High
Brunick A.L. et al., 1990 [7]	Level III	Low	High
Bucknor, A. et al., 2011 [18]	Level II	Good	High
Carter S. et al., 1996 [19]	Level II	Good	Low
Drabek, T. et al., 2010 [8]	Level II	Good	High
Gunasekera, P.C. et al., 1997 [20]	Level II	Good	Some
Hardison, S. A. et al., 2017 [21]	Level I	High	Low
Johnson, R. L. et al., 2013 [22]	Level I	High	Low
Kopka, A. et al., 2005 [26]	Level III	Good	Some
Man, T. et al., 2022 [23]	Level I	High	Low
Novak, C. B. et al., 1999 [27]	Level V	Low	High
Powell, B. J. et al., 1994 [28]	Level V	Low	High
Rudolph, R. et al., 2022 [29]	Level V	Low	High
Stellon, M.A. et al., 2016 [30]	Level V	Low	High
Stellon, M. et al., 2017 [31]	Level V	Low	High
Wilson, S. J. et al., 1996 [24]	Level I	Low	Some

## Data Availability

The data that support the findings of this consensus document are available from the corresponding author upon reasonable request.

## Supplementary Materials

The following supporting information can be downloaded at: https://www.mdpi.com/article/10.3390/jcm14217695/s1, Table S1: PRISMA 2020 Checklist: Hands Deserve Better: A Systematic Review on Surgical Glove Fit and Provider Performance.
